# A New Method of Regular and Constant Coarctation of Parts in Operations for Harelip

**Published:** 1862-11

**Authors:** 


A New Method of Regular and Constant Coarctation of Parts in Operations for Harelip.—*By Dr. Cooper.*—The use of the common harelip pin is objectionable on two grounds: first, the parts being held together in such a way that there is not uniform pressure upon all the cut surfaces, and, consequently, they are not in a favorable condition for healing by first intention throughout.

The pressure upon the parts immediately about the pins is sufficient, generally, to produce more or less absorption, while upon other portions of the cut surface it is not quite sufficient to produce that close approximation favorable to healing by first intention. This is a most serious impediment to success in operations upon young children, where firm union must take place early, else it will be broken up by the child crying.

The second objection is, that in making the figure-of-eight (8) suture there will be, often, at each extremity of the needles, more or less ulceration, which not only tends to favor the tearing out of the sutures, but is likely to cause deformity, to a greater or less extent. It is true we can often remove the needles before this result occurs, but then it is at the risk of causing a want of union in the margin of the lip. How often do we see in adults a little gap in the lower margin of the upper lip, occurring in consequence of a want of union, or from some slight separation after healing had taken place. This is owing to a want of proper support during the process of union of the parts; and in very young children we all know that it is sometimes particularly difficult to secure healing at all, because of the stretching of the lips in crying, tearing the parts asunder.

The method we now practice enables the surgeon to press all portions of the cut surfaces together equally, and yet so gently, that ulceration or sloughing will not be produced, in any case, by the pressure, and the tearing out of the sutures would not be at all likely to occur, inasmuch as the tissues do not become softened by inflammation, as happens, in all cases, when the pins are used.

With the use of the pins and figure-of-eight (8) suture it is impossible to keep the surfaces entirely even, but with the method now to be proposed, all this difficulty is obviated. It is as follows: A flat coil of lead wire, about two lines in diameter, is applied over the parts, after the cut surfaces are brought together, and large enough to reach from one-half to three-quarters of an inch on either side. A long curved needle, armed with a silver ligature, is then passed through the parts, from side to side, at various points, as well as different depths, some being very superficial and others entirely thro' the thickness of the lip, the ligature being carried around some portion of the coil every time it is carried from side to side. The coil is made by bending the wire, five or six times, around a pen-holder or pencil, having the different portions of it a line or two apart, according to the amount of space to be covered by it.

The coil being fastened to its place, it is then curved, or otherwise shaped, so as to place the lip in the best possible position, as respects the face, when the parts will be retained, the lips and wire being as easily molded as putty. A piece of lint, wet in an evaporating lotion, composed of one part of alcohol and ten of water, is then applied to the part, and adhesive plasters applied, reaching from one ear to the other, when the dressing is complete.

The coil and silver ligatures should, generally, remain in their places from one to three weeks, according to the rapidity of the healing of the parts. But as they confer so little inconvenience by their presence, it is always well to let them remain until there can be no danger of a separation of the parts.

There is scarcely ever found any ulceration worth mentioning. Sometimes a very little will be found under some portion of the coil, where it presses rather strongly, but none in the track of the ligature. The openings whence they were taken will be entirely closed the next day after their removal, so little is the irritation produced by their presence in the tissues.

Prior to applying the coil of lead, the wound may be closed by three or four stitches, made with very fine thread, the needle being introduced to but very little depth.—*San Francisco Medical Press.*

Complete Reproduction of the Temporo-Maxillary Articulation.—*By Dr. Cooper.*—The little girl from whom was removed the parotid and sub-maxillary glands, mentioned in the Press, some months since, has entirely recovered.

In that case the tumor involving these glands could not be removed without either taking away a portion of the jaw or destroying temporarily the temporo-maxillary articulation, so that the jaw on that side might be moved forwards out of its place, thus giving room for the extraction of the tumor. The latter method was adopted, and, as was anticipated at the time, a complete reproduction of the joint has taken place.

The wound is entirely cicatrized, and the patient moves the jaw almost as well as ever.

The free-opening of joints and having all their structures reproduced, is getting to be considered as a small matter in California.—*San Francisco Medical Press.*

